# Silver microsphere doping porous-carbon inspired shape-stable phase change material with excellent thermal properties: preparation, optimization, and mechanism

**DOI:** 10.1038/s41598-020-77901-6

**Published:** 2020-11-30

**Authors:** Junwei Zhang, Yan Chen, Zeguang Nie, Zhengshou Chen, Junkai Gao

**Affiliations:** School of Naval Architecture and Maritime, Zhejiang Ocean University, Zhoushan, 316022 China

**Keywords:** Solar energy, Energy storage

## Abstract

In this study, silver microspheres (SMS) were introduced into cotton stalk porous-carbon (CSP) to prepare silver microsphere doping porous-carbon (SMS-CSP), and then SMS-CSP was used as the matrix of polyethylene glycol (PEG) to synthesize shape-stable phase change material of PEG/SMS-CSP. It was found that the introduction of SMS into CSP could not only greatly improve the loading capacity of the porous-carbon for PEG, but also could increase the thermal conductivity of PEG/SMS-CSP. Additionally, the method of introducing SMS into porous-carbon had the advantages of environmental protection and simple operation. Moreover, the raw material of cotton stalk is a kind of agricultural waste, which has the merits of wide source, low price and easy to obtain. Furthermore, in the preparation of cotton stalk porous-carbon, with the increase of pyrolysis temperature the thermal conductivity of PEG/SMS-CSP could be enhanced significantly. The mechanism about the enhancement of thermal conductivity was clarified, which could provide more basic theory for the study about the thermal conductivity of shape-stable phase change materials (ss-PCMs) based on porous-carbon.

## Introduction

On the basis of rapid economic development, the imbalance between the energy consumption and supply makes people aware of the importance of renewable energy. Energy storage technology is of great significance for solving energy shortage problems. Up to now, energy storage technologies could be classified according to energy storage medium, which could be divided into mechanical energy storage, latent heat energy storage, electrical energy storage and chemical energy storage^1^. Latent heat energy storage is to store or release energy by absorbing or releasing heat in the process of phase change by using phase change materials (PCMs). The reusability, high energy storage density and high heat recovery rate of the PCMs had helped to attract much attention^2^. PCMs are widely used in varied fields, such as power peak-load shifting, building energy saving, industrial waste heat utilization and solar energy utilization^3^. PCMs could be classified as organic PCMs and inorganic PCMs on the basis of chemical structure. Among them, organic PCMs mainly include polyethylene glycol (PEG), paraffin, higher fatty acids, polyolefin, etc., which possess a series of advantages such as good fixed molding, less prone to phase separation, and small corrosion. However, the organic PCMs still have the disadvantages of seepage and low thermal conductivity, which hinder their practical application.

Fixing the organic PCMs in porous materials to synthesize shape-stable phase change materials (ss-PCMs) is an effective way to solve the above problems^4^. Many porous materials, such as mesoporous silica^5^, diatomaceous earth^6^, expanded graphite^7^, graphene^8^, aerogel^9^, metal foam^10^, metal organic skeleton^11^ and biochar^12^ were used as the support materials. Biochar is a carbon-rich material that could be produced from a variety of organic waste materials such as agricultural waste and municipal sewage sludge by pyrolysis of biomass under anoxic or anaerobic conditions. Biochar is receiving more and more attention due to its unique characteristics such as high carbon content, large specific surface area and stable structure^13,14^. Wu et al.^15^ utilized biochar as a soil improvement agent to increase the available phosphorus content in the saline-alkali soil and reduce the leaching of phosphorus in the soil.

Additionally, the leakage of organic PCMs could be avoided by using biochar as carrier to prepare ss-PCMs. For example, Jeon et al.^14^ utilized organic-type PCMs as core material and rice husk biochar as porous supporting material to design bio-based ss-PCMs, and the obtained ss-PCMs exhibited high thermal stability. Chen et al.^16^ prepared ss-PCMs with biochar as the matrix and polyethylene glycol as the core material by vacuum impregnation method, and the results showed that the ss-PCMs had favorable thermal stability and good thermal reliability. However, the thermal conductivity of ss-PCMs prepared using biochar as the carrier still needed to be improved. Recent studies found that adding high thermal conductive nanoparticles to ss-PCMs is an effective method to enhance their thermal conductivities. Oya et al.^17^. utilized graphite to fix erythritol for the preparation of high thermal conductive ss-PCMs by adding nickel particles. Cheng et al.^18^ designed ss-PCMs of tetradecanol/expanded perlite, and the thermal conductivity of tetradecanol/expanded perlite was improved by adding Cu powder. However, there are still some defects in the preparation of ss-PCMs with improved thermal conductivity by adding high thermal conductive nanoparticles, such as high cost, complex process and easy to pollute the environment.

In order to solve the above problems, our research group developed an in-situ reduction method for the preparation of ss-PCMs with improved thermal conductivity based on carbon material^19^, and in that study, mesoporous carbon was used to adsorb copper ions firstly, and then copper ions were reduced to copper microspheres by in-situ reduction at high temperature. Then mesoporous carbon doping with copper microspheres was obtained, which was used as the support of organic PCM to prepare ss-PCMs with improved heat conduction performance. This method has the advantages of simple operation, environment-friendly and low cost. Additionally, the copper microspheres synthesized by in-situ reduction method could be firmly combined on the surface of mesoporous carbon, which could prevent the deposition of copper microspheres in the phase transformation process of ss-PCMs and increase the thermal conductive stability. However, there are still some disadvantages about this thermal conductivity enhancement method, such as low loading content of core material and relatively small increase range of the thermal conductivity.

Aim to further improve the loading capacity and thermal conductivity of ss-PCMs that was prepared by using in-situ reduction strategy, in this study, the cotton stalk, which is a kind of agricultural waste and has the advantages of wide source, low price, easy to obtain, high carbon content, etc., was used as the raw material to prepare cotton stalk porous-carbon (CSP)^20,21^, and then silver microspheres were synthesized by in-situ reduction strategy on the surface of CSP. The ss-PCMs (PEG/SMS-CSP) was prepared by using PEG as core material and silver microsphere doping cotton stalk porous-carbon (SMS-CSP) as the carrier. The thermal properties of PEG/SMS-CSP were studied in detail.

In this paper, it was found that the introduction of SMS into CSP could not only greatly improve the loading capacity of the porous-carbon for PEG, but also could increase the thermal conductivity of the ss-PCMs of PEG/SMS-CSP. Moreover, the method of introducing silver microspheres into porous-carbon had the advantages of environmental protection and simple operation. More importantly, to the best of our knowledge, there was no study report about the effect of different pyrolysis temperature on the properties of ss-PCMs used porous-carbon as the supporter, and in this study, it was found that with the increase of pyrolysis temperature of cotton stalk porous-carbon, the thermal conductivity of PEG/SMS-CSP could be enhanced significantly, and the enhancement mechanism was clarified.

## Materials and methods

### Materials

The cotton stalks were obtained from Chuzhou, Anhui, China. Silver nitrate was obtained from Shanghai Zhanyun Chemical Co, Ltd, and ethanol was obtained from Chemical Reagent Sinopharm Holding Co, Ltd. Polyethylene glycol (Mn = 4000) was obtained from Aladdin Reagent Co, Ltd.

### Preparation of PEG/SMS-CSP

**Figure 1:**
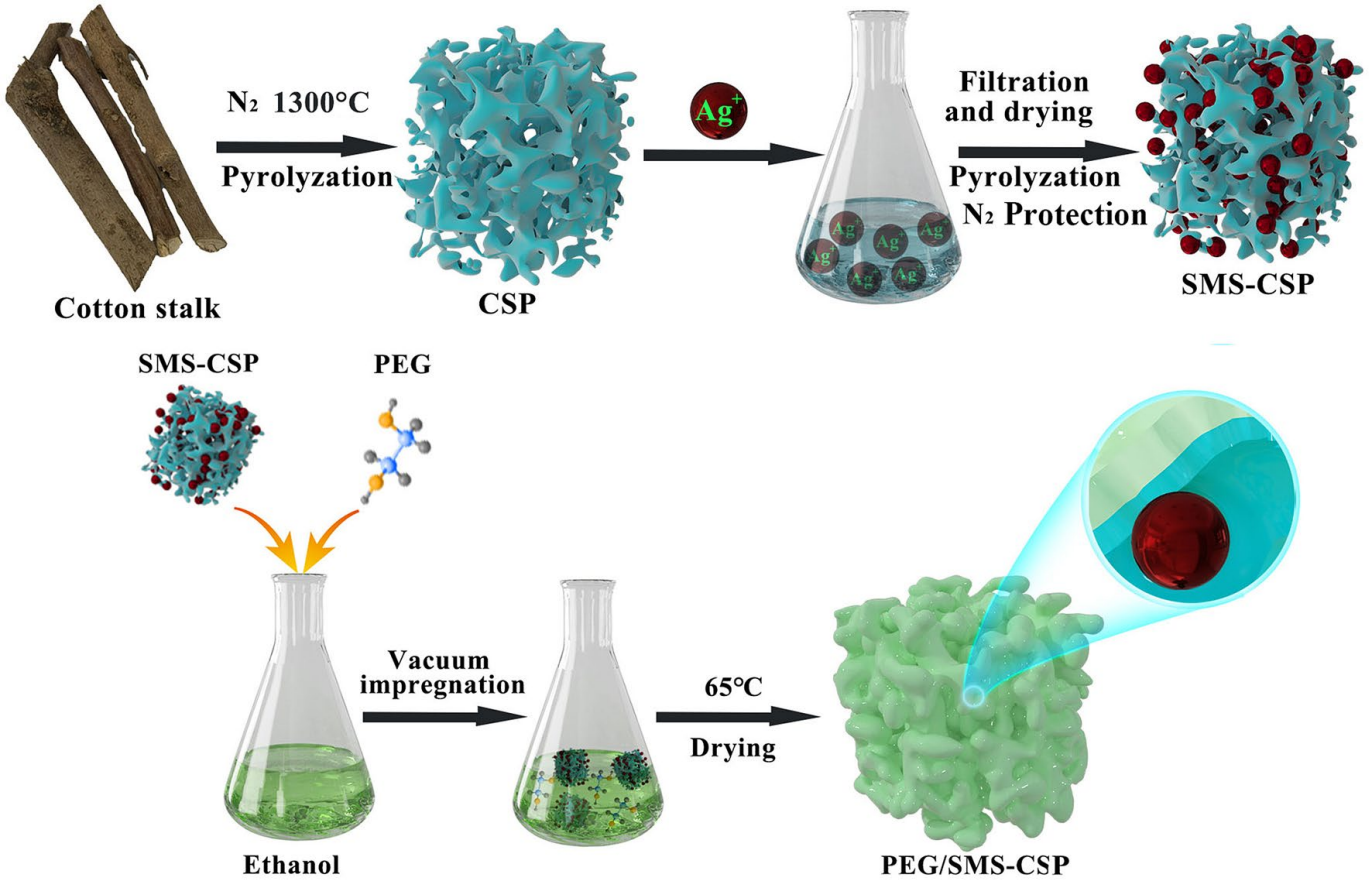
Schematic diagram for the synthesization of PEG/SMS-CSP.

The schematic diagram for the synthesization of PEG/SMS-CSP is shown in Fig. 1. Firstly, CSP was prepared by high temperature pyrolysis (1300 °C) using cotton stalk as the raw material. Then a certain mass of CSP was added into 100 ml silver nitrate solution with concentration of 800 mg/L and oscillated at 25 °C for 4 h. After that, the suspension was filtrated, and the obtained product was dried at 50 °C for 12 h. The dried products were put into the tubular furnace and heated to a certain temperature in nitrogen atmosphere. Finally the obtained product was SMS-CSP.

The PEG/SMS-CSP with different PEG contents was prepared by vacuum impregnation method. PEG solutions with different concentrations were synthesized by adding a certain amount of PEG (0.16 g, 0.2 g, 0.23 g) into 15 ml anhydrous ethanol. After that a certain amount of SMS-CSP (0.16 g, 0.13 g, 0.1 g) were added into the above solutions. The above suspension was agitated in vacuum condition for 1 h, and then continuously stirred in a constant temperature water bath for 1 h at 45 °C. Finally, the black solid product was dried at 50 °C for 12 h, and three kinds of ss-PCMs were prepared, which were named as 50% PEG/SMS-CSP, 60% PEG/SMS-CSP and 70% PEG/SMS-CSP. For comparison, the 50% PEG/CSP and 60% PEG/CSP without SMS were prepared by using the same synthesis procedure as for 50% PEG/SMS-CSP and 60% PEG/SMS-CSP.

### Characterization

The morphologies of CSP, SMS-CSP and PEG/SMS-CSP were investigated by scanning electron microscopy (SEM, Quanta FEG-250, FEI, America). Spectroscopic analysis was performed by using Fourier transform infrared spectrometer (FT-IR, Bruker VECTOR22), and the crystalloid phase of the samples was tested by wide-angle X-ray diffraction (XRD, DX-2700, SHL-2, Thermo Scientific).Thermo gravimetric analysis was utilized to test the thermal stability of PEG and PEG/SMS-CSP in N_2_ atmosphere from 50 to 500 °C with heating rate of 10 °C/min. The specific surface area and pore volumes of the SMS-CSP and CSP was evaluated through Brunauer–Emmett–Teller (BET) method (Nova2000e, Quantum, America).The enthalpy and phase transition temperature of PEG, CSP and PEG/SMS-CSP samples were accurately measured by utilizing differential scanning calorimetry (DSC, Q200, NETZSCH, Germany), which were carried out in the temperature range of 0 °C to 100 °C by using high purity nitrogen as the protective gas, and the heating and cooling rate was 10 °C/min. X-ray photoelectron spectroscopy (XPS, ESCALAB 250Xi KAlpha, Thermo Fisher) was used to record the spectra of CSP and SMS-CSP. The transient plane source method (TPS, CTPS-2500, FRD, Tianjin, China) was used to measure the thermal conductivities of PEG and PEG/SMS-CSP.

## Results and discussions

### Surface morphology

**Figure 2:**
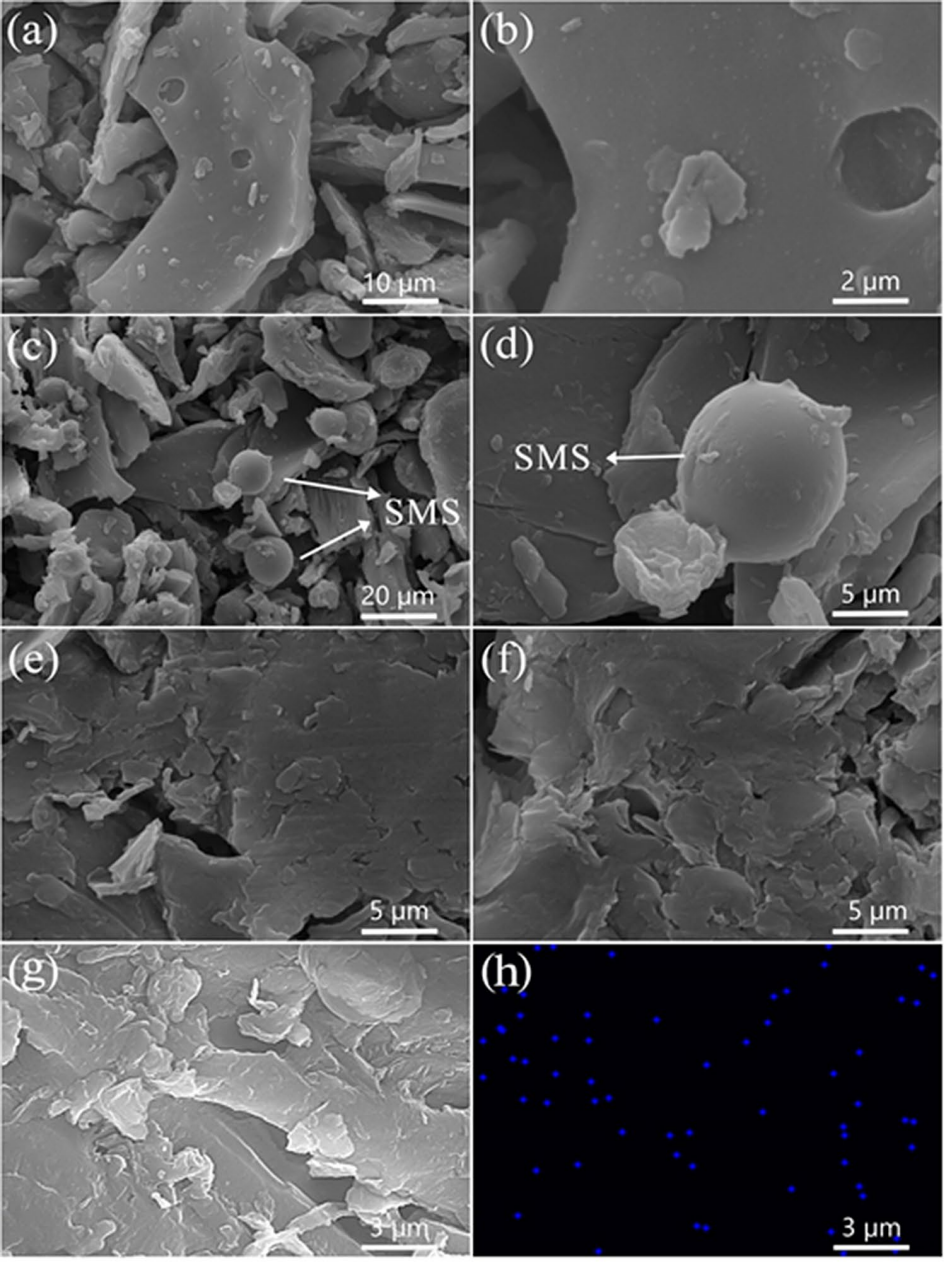
SEM images of CSP (**a**,**b**), SMS-CSP (**c**,**d**), 70% PEG/SMS-CSP (**e**–**g**). EDS elemental mapping analysis of the 70% PEG/SMS-CSP (**h**).

The SEM images of CSP, SMS-CSP and 70% PEG/SMS-CSP are shown in Fig. 2. Figure 2a,b are the SEM images of CSP. The porous-carbon of CSP prepared by pyrolysis method had rough surface, which were beneficial for adsorbing organic PCMs. SEM images of SMS-CSP are shown in Fig. 2c,d, and it could be seen that silver ions were reduced to silver microspheres and silver microspheres with different sizes were distributed on the surface of CSP randomly. Figure 2e–g are the SEM images of 70% PEG/SMS-CSP, and the surface morphology of 70% PEG/SMS-CSP showed a closely dense structure after introducing PEG into the pores of SMS-CSP, indicating that the PEG molecules were adsorbed in the SMS-CSP completely, which was attributed to the Van der Waals force and capillary force between the PEG and SMS-CSP^22^. In order to directly and clearly observe the distribution characteristics of silver element, the element surface distribution of 70% PEG/SMS-CSP was tested, and Fig. 2h exhibits the EDS elemental mapping of 70% PEG/SMS-CSP. It could be seen from the picture that the distribution of Ag element was irregular, and the quantity of silver element was small, which was due to the small ratio of silver mass to the total mass of the sample.

### Physical properties analysis

**Figure 3:**
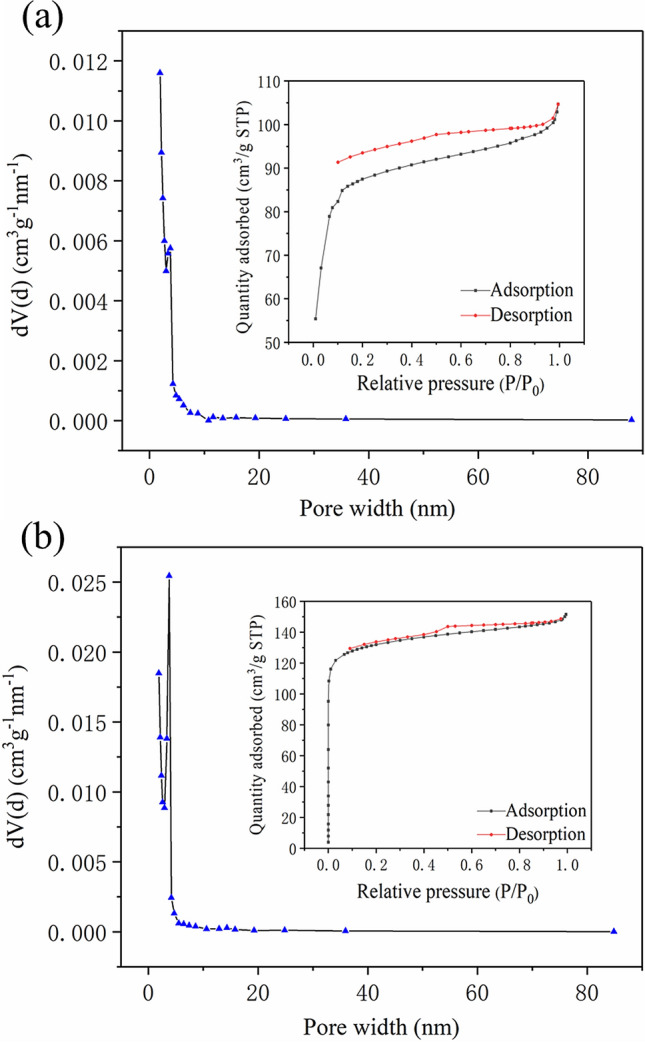
(**a**) N_2_ adsorption–desorption isotherms and pore size distribution of CSP (**b**) N_2_ adsorption–desorption isotherms and pore size distribution of SMS-CSP.

The pore size distribution diagram, nitrogen adsorption and desorption isotherms of CSP and SMS-CSP are shown in Fig. 3. Figure 3a shows that the pore size distribution of CSP focused on 1.8–2.0 nm and 2.0–15.1 nm. The specific surface area of CSP was 344.23 m^2^/g, and its pore volume was 0.12 cm^3^/g. It could be seen from Fig. 3b that the pore volume of SMS-CSP was 0.23 cm^3^/g, and its specific surface area was 509.04 m^2^/g. It could be seen that the specific surface area of CSP was 344.23 m^2^/g, which was much less than that of SMS-CSP. Compared with CSP, the higher micro/mesopore proportions were observed for SMS-CSP, which had a significant impact on PCM encapsulation. During the phase transition process, a large micro/mesopore proportion could prevent the organic PCMs from leakage because of the strong capillary force^23^. Therefore, introducing silver microspheres into CSP could increase its specific surface area, which could enhance the loading capacity of SMS-CSP for organic PCMs.

### Chemical compatibility analysis

**Figure 4:**
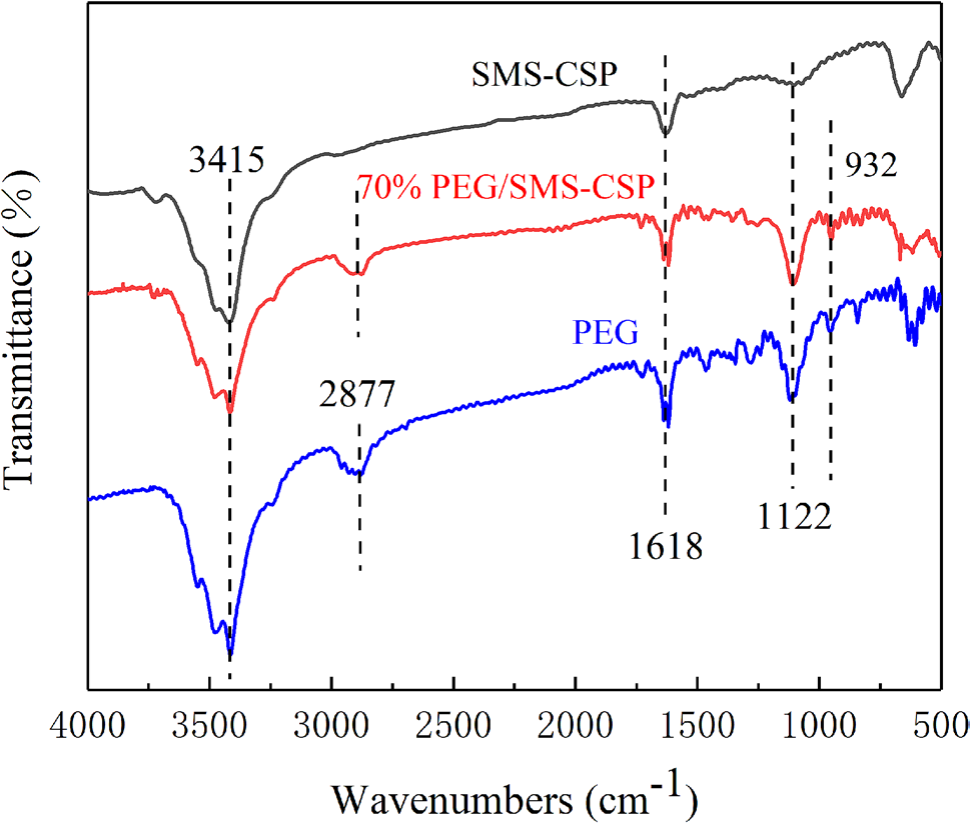
FTIR spectra of SMS-CSP, 70% PEG/SMS-CSP and PEG.

The chemical stability and chemical compatibility of the samples could be studied by the FT-IR spectroscopy. Figure 4 depicts the FT-IR spectroscopy curves of SMS-CSP, 70% PEG/SMS-CSP, and PEG. The peak at 1618 cm^−1^ was attributed to the stretching vibration of –OH^24^. The absorption peak at 3415 cm^−1^ was attributed to the stretching vibration peak of –OH^19^. The absorbing peak at 2877 cm^−1^ was because of the symmetrical stretching vibration of CH_2_^26^. The adsorption band at 1122 cm^−1^ was because of the stretching vibration of C–O, and the absorption peak at 932 cm^–1^ was the in-plane deformation vibration of C–O^16^. For the 70% PEG/SMS-CSP, all of the characteristic absorption peaks of PEG and SMS-CSP could be found in the spectra of 70% PEG/SMS-CSP, and no new peak appeared. Therefore, it was only physical interaction in the preparation of 70% PEG/SMS-CSP, which showed that the SMS-CSP developed in this study was ideal carriers for PEG immobilization, and the as-prepared ss-PCMs of 70% PEG/SMS-CSP had favorable compatibility and chemical stability.

**Figure 5:**
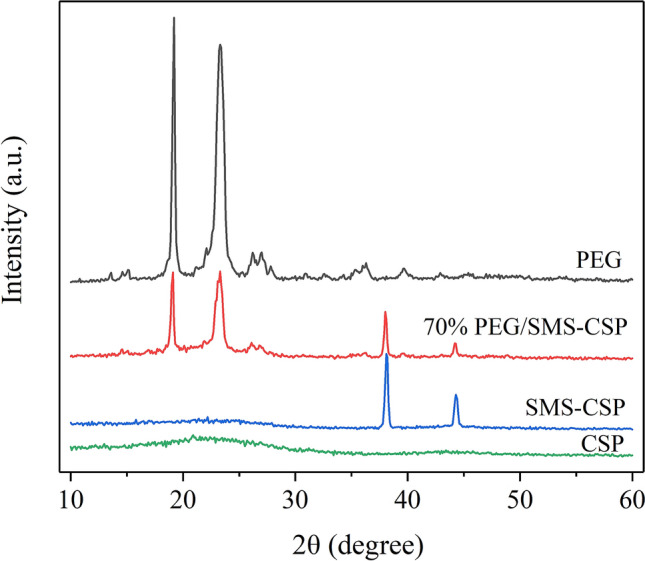
The XRD patterns of CSP, SMS-CSP, 70% PEG/SMS-CSP and PEG.

XRD could be used to measure the chemical compatibility and crystallization properties of the samples. The XRD patterns of CSP, SMS-CSP, 70% PEG/SMS-CSP and PEG are shown in Fig. 5. The CSP indicated a relatively amorphous structure with two broad peaks, which reflected the (0 0 2) and (1 0 0) planes of graphitic carbon materials at approximately 2θ = 23.40° and 44.20°, respectively^23^. For the pattern of SMS-CSP and 70% PEG/SMS-CSP, two different diffraction peaks were found at 38.10° and 44.30°, which represented the crystal surface of the silver monomer. This result indicated that silver microspheres were successfully introduced into CSP. Two typical peaks in PEG pattern appeared at 19.20° and 23.30°, and these two peaks could also be found in the 70% PEG/SMS-CSP pattern, which had the same shape, indicating that PEG was fixed in SMS-CSP successfully. Additionally, even when PEG was fixed in SMS-CSP, PEG could still maintain excellent crystalline properties and its crystal structure was not damaged. However, the diffraction peak of 70% PEG/SMS-CSP was significantly smaller than that of pristine PEG, which meant that the crystallinity of 70% PEG/SMS-CSP was lower than that of pristine PEG. It might be due to that the pore structure of SMS-CSP limited the crystallinity of PEG molecules in 70% PEG/SMS-CSP, and this result could be attributed to the Van der Waals force and capillary force between SMS-CSP and PEG molecules. In addition, no new peak appeared in the XRD pattern of 70% PEG/SMS-CSP, which indicated that there was no chemical reaction in the preparation process of 70% PEG/SMS-CSP.

### XPS analysis

**Figure 6:**
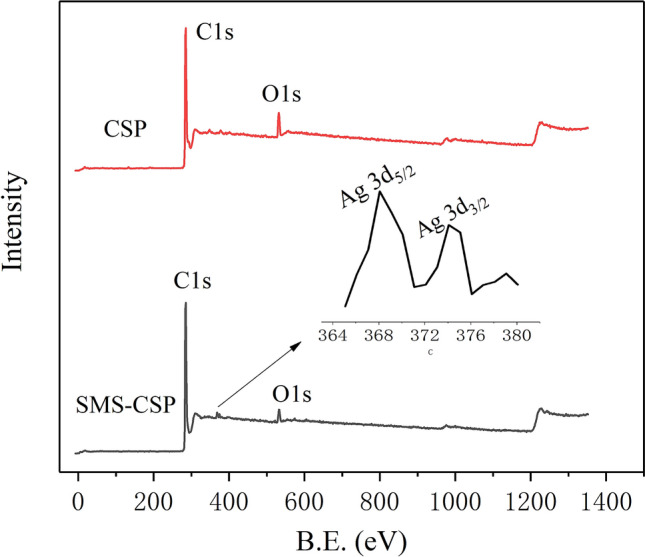
XPS patterns of CSP and SMS-CSP.

The elemental composition of CSP and SMS-CSP was determined by XPS. The XPS curves of CSP and SMS-CSP were shown in Fig. 6, and C, O and Ag were the main elemental components of SMS-CSP. As can be seen from the XPS curve of SMS-CSP, the binding energies of C 1s and O 1s were 284.38 eV and 533.28 eV, respectively. Due to the dissipation of oxygen in the process of reducing silver ions to silver microspheres, the carbon–oxygen ratio of CSP was reduced after the introduction of SMS. In addition, compared with CSP, the Ag 3d XPS spectrum of 70% PEG/SMS-CSP exhibited two obvious peaks corresponding to Ag 3d_3/2_ (374.28 eV) and Ag 3d_5/2_ (368.23 eV), indicating that the successful introduction of SMS into CSP.

### Leakage tests of PEG and PEG/SMS-CSP composites

**Figure 7:**
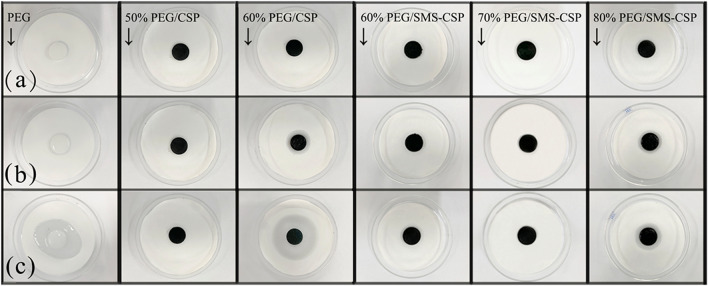
Leakage test photographs of PEG and different theoretical PEG contents of PEG/CSP and PEG/SMS-CSP. (**a)** Pictures of the samples at room temperature; (**b**) Pictures of samples at 65 °C for 5 min; (**c**) Pictures of samples at 65 °C for 10 min.

Leakage experiments, which were used to determine and compare the shape stability of pure PEG, 50% PEG/CSP, 60% PEG/CSP, 60% PEG/SMS-CSP, 70% PEG/SMS-CSP and 80% PEG/SMS-CSP, were carried out to check whether they would leak by putting them into a baking oven at 65 °C for 10 min, and the results are exhibited in Fig. 7. When the temperature reached 65 °C, which was higher than the melting temperature of PEG, the pure PEG, 60% PEG/CSP and 80% PEG/SMS-CSP leaked within 5 min and began to diffuse on the filter paper. After 10 min, the diffusion phenomenon was more obvious. However, 60% PEG/SMS-CSP, 70% PEG/SMS-CSP and 50% PEG/CSP could keep its shape even after 10 min without any leakage, which might be attributed to PEG being fixed by weak hydrogen bonds of functional groups and absorbed by capillary force^23^. According to the results, the maximum PEG loading amount in PEG/CSP was 50%, and however, the maximum PEG loading amount in PEG/SMS-CSP could reach 70%. This result could be attributed to that when SMS was introduced into CSP, the surface area of SMS-CSP was increased obviously compared to that of CSP, which could also be verified by the study results of BET tests. Therefore, the adsorption capacity of SMS-CSP was much larger than that of CSP, and the loading capacity of SMS-CSP was improved significantly.

### Thermal stability analysis

**Figure 8:**
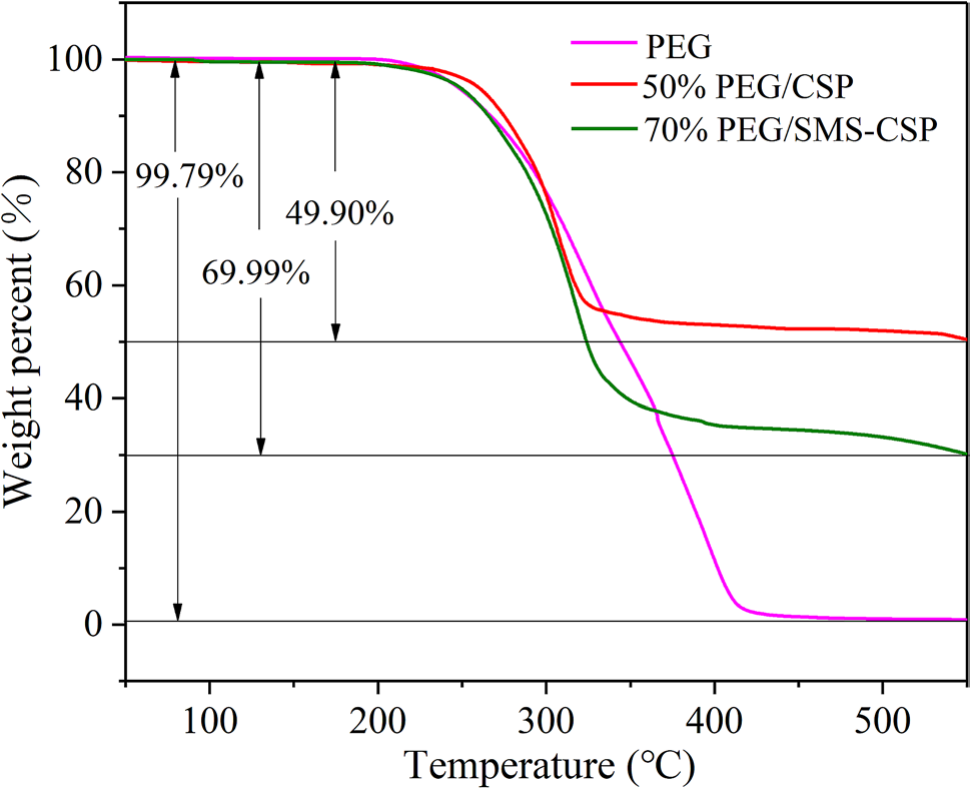
TGA curves of PEG, 50% PEG/CSP and 70% PEG/SMS-CSP.

Figure 8 exhibits the TGA thermograms of PEG, 50% PEG/CSP and 70% PEG/SMS-CSP. According to the TGA curve of PEG, the pure PEG almost did not show degradation below 200 °C. The weight loss percent of PEG was about 99.79%, and the 0.21% residues might be caused by operating errors or the impurities of PEG. For the samples of 50% PEG/CSP, it decreased rapidly in the temperature range of 250–330 °C, and the total weight loss rate was 49.90%. As for the TGA thermogram of 70% PEG/SMS-CSP, there was a slight weight loss before the temperature of 150.00 °C and the corresponding weight loss percent was 0.49%, which was owing to the evaporation of water adsorbed on the surface of 70% PEG/SMS-CSP. With the increase of temperature, the PEG in 70% PEG/SMS-CSP began to evaporate and decompose, and the mass of 70% PEG/SMS-CSP decreased significantly when the temperature went from 228.68 to 342.12 °C, which was caused by the thermal degradation of PEG in the composite of 70% PEG/SMS-CSP. The total weight loss percent of 50% PEG/CSP and 70% PEG/SMS-CSP was 49.90% and 69.99%, respectively, which was slightly less than 50% and 70.00%, and this result might be caused by the operation error. Additionally, the decomposition rate of PEG in 70% PEG/SMS-CSP was higher than that of pristine PEG. This result was due to the higher thermal conductivity of 70% PEG/SMS-CSP than that of the pristine PEG, which accelerated the thermal decomposition of PEG in 70% PEG/SMS-CSP, thus reducing the decomposition temperature of PEG^25^. However, according to the study results, the 70% PEG/SMS-CSP still had high-temperature resistance and excellent thermal stability.

### Thermal properties analysis

**Figure 9:**
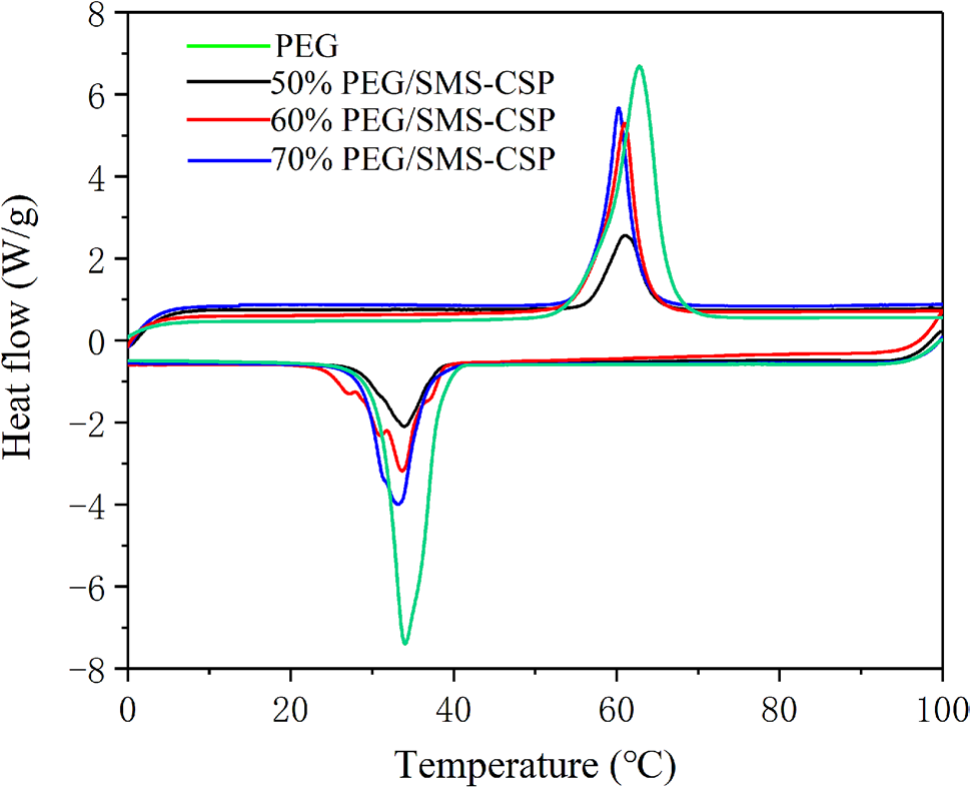
The DSC patterns of 50% PEG/SMS-CSP, 60% PEG/SMS-CSP, 70% PEG/SMS-CSP and PEG.

The DSC curves of PEG, 50% PEG/SMS-CSP, 60% PEG/SMS-CSP and 70% PEG/SMS-CSP are shown in Fig. 9, and these DSC curves were similar, which meant that in the phase change process, the PEG mainly served as the latent thermal energy storage material^26^. Table 1 exhibits the thermal parameters gained from the DSC test results, which contained the freezing temperature (T_f_), freezing latent heat (ΔH_f_), melting temperature (T_m_), and melting latent heat (ΔH_m_). The study results proved that the SMS-CSP hardly had influence on the phase transformation temperature of PEG. For instance, the freezing and melting temperatures of pristine PEG were 62.80 °C and 34.00 °C, respectively, and for the 70% PEG/SMS-CSP, the values were 60.90 °C and 33.20 °C, and the similar study results were widely mentioned in the literatures, such as GO/PEG^27^ and SiO_2_/PEG^28^ composite PCMs. In Table 1, the freezing and melting latent heats of PEG were 223.4 J/g and 207.4 J/g, respectively. Additionally, the 70% PEG/SMS-CSP had melting and freezing enthalpies of 122.9 J/g and 114 J/g, respectively. As for the PEG/SMS-CSP composite, because of the addition of SMS-CSP, the percentage content of PEG in the composite decreased, and therefore, the melting and freezing enthalpies of PEG/SMS-CSP were lower than that of the pure PEG. Moreover, the practical melting and solidifying enthalpies of PEG/SMS-CSP composites were lower than their theoretic values, which indicated that the crystallization characteristics of PEG molecules in the PEG/SMS-CSP composites were limited, and the regularities of crystal line regions of PEG molecules declined and the lattice defects increased, which resulted in the decrease of practical phase change enthalpies of PEG/SMS-CSP composites^29^.

**Table 1:** The thermal properties of composite PCMs.

Sample	Melting		Freezing	
	T_m_ (°C)	△H_m_ (kJ/kg)	T_f_ (°C)	△H_f_ (kJ/kg)
PEG	62.80	223.40	34.00	207.40
50% PEG/SMS-CSP	60.86	54.44	35.80	50.97
60% PEG/SMS-CSP	60.20	111.60	33.70	103.10
70% PEG/SMS-CSP	60.90	122.90	33.20	114.00

**Figure 10:**
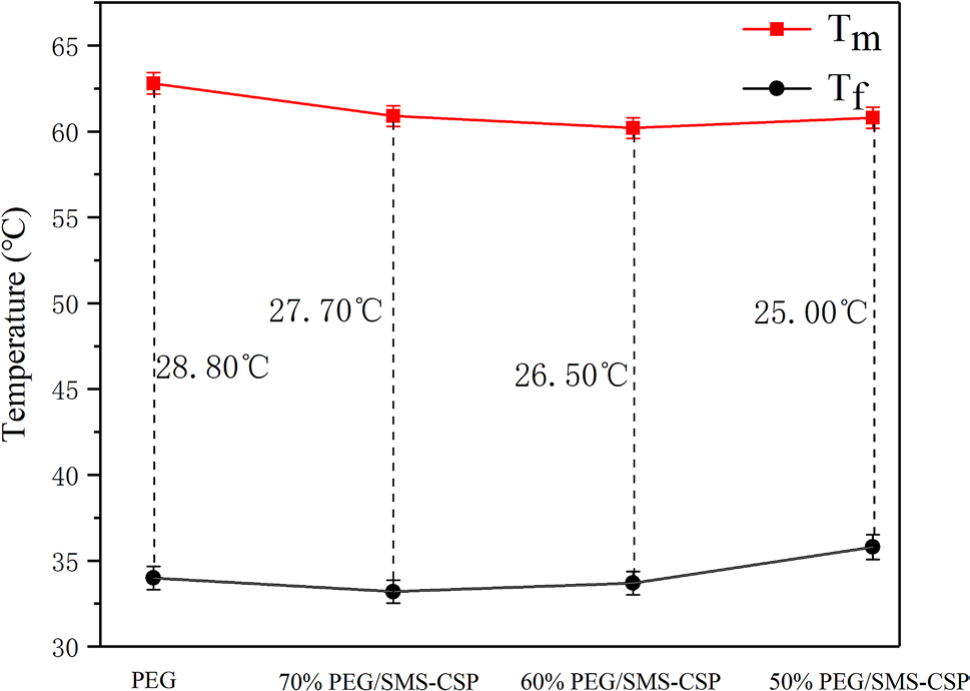
Supercooling phenomenon of 50% PEG/SMS-CSP, 60% PEG/SMS-CSP, 70% PEG/SMS-CSP and PEG.

The melting points of 50% PEG/SMS-CSP, 60% PEG/SMS-CSP, and 70% PEG/SMS-CSP were decreased slightly in comparison with that of pure PEG. The changes in melting points were representation of the strength of interactions between the supporting materials and PEG^31^. For a weak interaction between porous materials and organic PCMs, the melting point would decline, while a strong interaction would result in an increase of phase change temperature. Additionally, the PEG molecules might interact with the porous carbon through hydrogen bonding and capillary force, which led to lower melting point^30^. The difference value of T_f_ and T_m_ is expressed as the supercooling temperature (ΔT)^31^. As can be seen from Fig. 10, the supercooling temperature of the pure PEG was 28.80 °C, and the values of the PEG/SMS-CSP composites were decreased. For example, the supercooling temperature of 50% PEG/SMS-CSP was 25.00 °C, which was lower than that of the pure PEG. Therefore, the preparation of PEG/SMS-CSP composites could reduce their supercooling extent, which was beneficial for the practical applications of PEG/SMS-CSP composites.

### Thermal reliability

**Figure 11:**
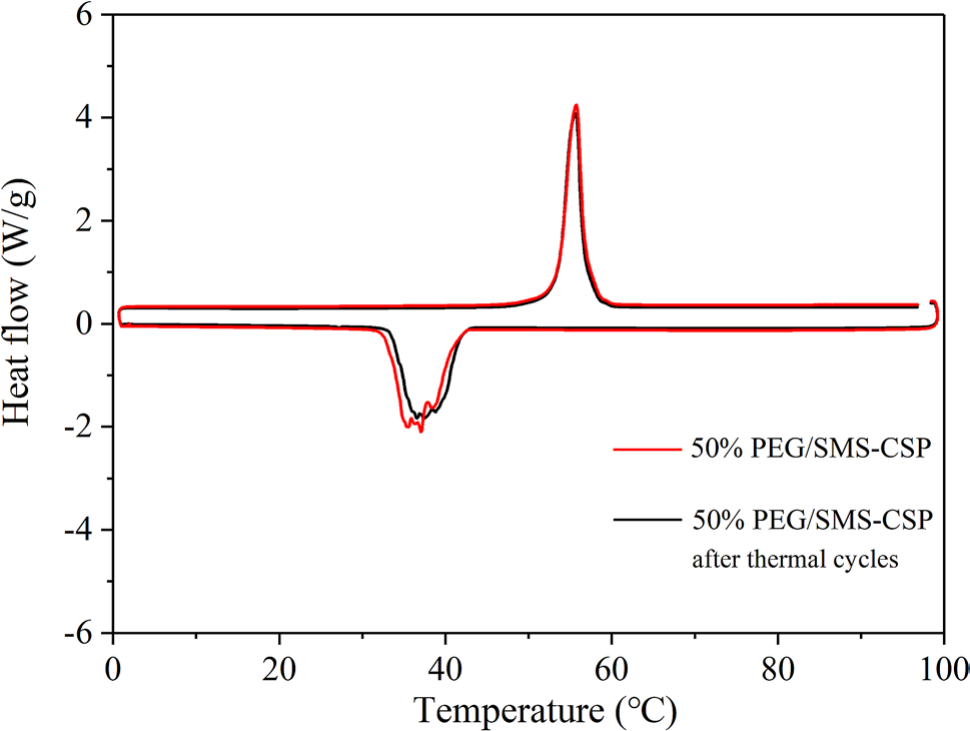
DSC patterns of 50% PEG/SMS-CSP before and after thermal cycling tests.

**Figure 12:**
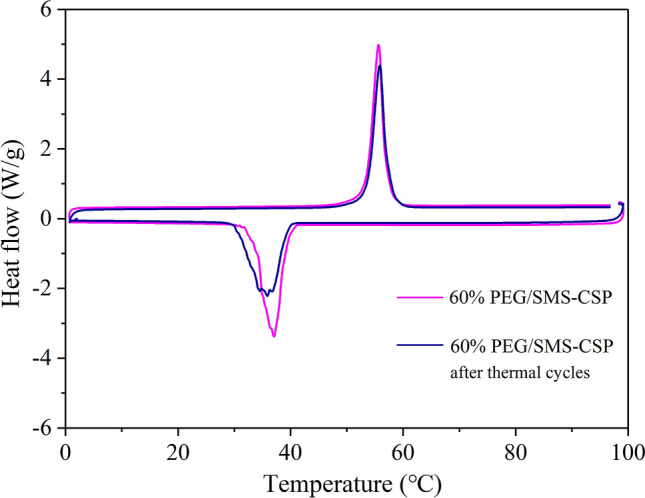
DSC patterns of 60% PEG/SMS-CSP before and after thermal cycling tests.

**Figure 13:**
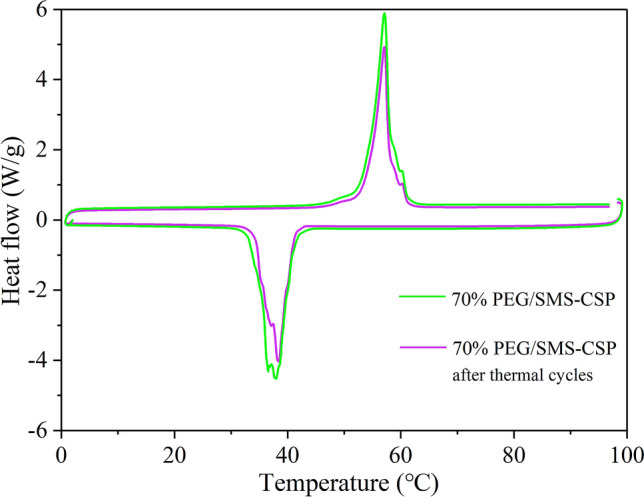
DSC patterns of 70% PEG/SMS-CSP before and after thermal cycling tests.

In order to study the thermal reliability of PEG/SMS-CSP, the DSC tests for 50% PEG/SMS-CSP, 60% PEG/SMS-CSP and 70% PEG/SMS-CSP before and after 100 thermal cycles were carried out, and the results are shown in Figs. 11, 12 and 13. The results showed that 100 thermal cycle tests had little effect on the phase transition temperature of 50% PEG/SMS-CSP, 60% PEG/SMS-CSP and 70% PEG/SMS-CSP. Additionally, after 100 times thermal cycling, the melting enthalpies of 50% PEG/SMS-CSP, 60% PEG/SMS-CSP and 70% PEG/SMS-CSP decreased by 0.16 J/g, 2.50 J/g and 1.00 J/g, respectively, and their freezing enthalpies also decreased by 0.20 J/g, 1.88 J/g and 0.40 J/g, respectively. Hence, the enthalpies the PEG/SMS-CSP composites exhibited small changes after 100 times thermal cycling and the trend of the DSC curves remained almost the same. The slight decrease of the enthalpies might be caused by the evaporation of a small amount of PEG. The above results meant that no leakage was occurred with the PEG/SMS-CSP composites, which were endowed with excellent thermal cycling stability. Therefore, the PEG/SMS-CSP developed in this study had good thermal reliability and favorable application prospects.

### Thermal conductivity

**Figure 14:**
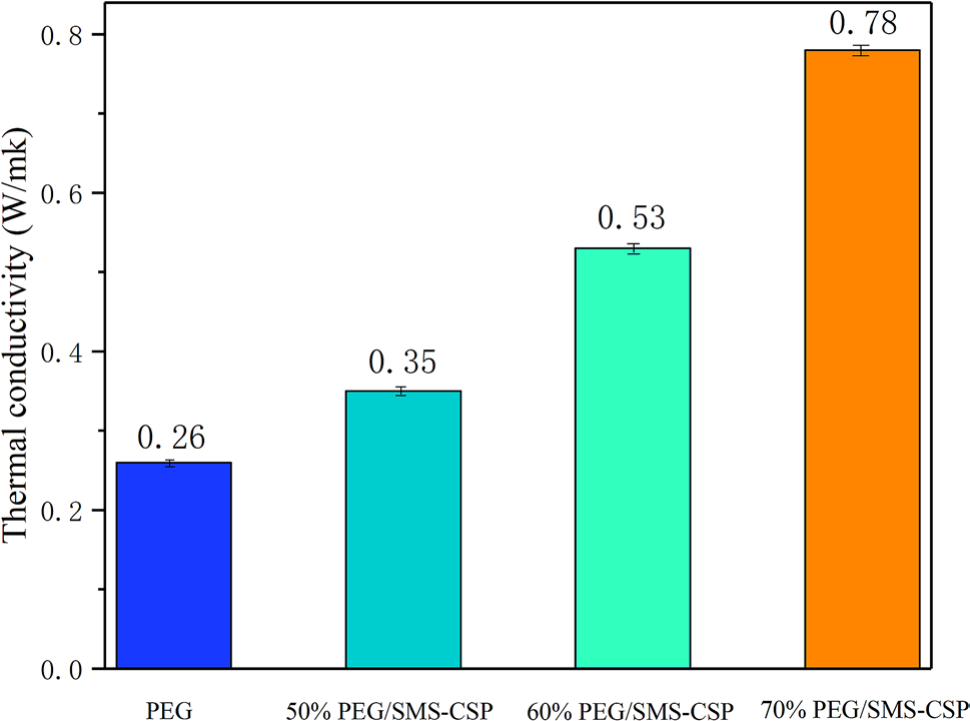
Thermal conductivity of PEG, 50% PEG/SMS-CSP, 60% PEG/SMS-CSP, and 70% PEG/SMS-CSP.

Figure 14 shows the thermal conductivity of PEG, 50% PEG/SMS-CSP, 60% PEG/SMS-CSP and 70% PEG/SMS-CSP, which were 0.26 W/(m k), 0.35 W/(m k), 0.53 W/(m k) and 0.78 W/(m k), respectively. It could be seen from Fig. 14 that the thermal conductivity of PEG/SMS-CSP composites improved with the increase of PEG content. This result might be attributed to the carbon like structure of CSP, which contained a large number of micro/mesopores. During the preparation of ss-PCMs by vacuum impregnation, PEG could be easily adsorbed on the surface and channel of SMS-CSP. With the increase of PEG content, the channel of SMS-CSP could be completely filled by PEG, and the PEG/SMS-CSP was more compact. When the content of PEG was low, a small amount of PEG was dispersed in CSP, and parts of the channel of SMS-CSP were still filled with air, which was not conducive to the formation of thermal conductivity path. Therefore, with the increase of PEG content, the thermal conductivity of PEG/SMS-CSP enhanced.

**Figure 15:**
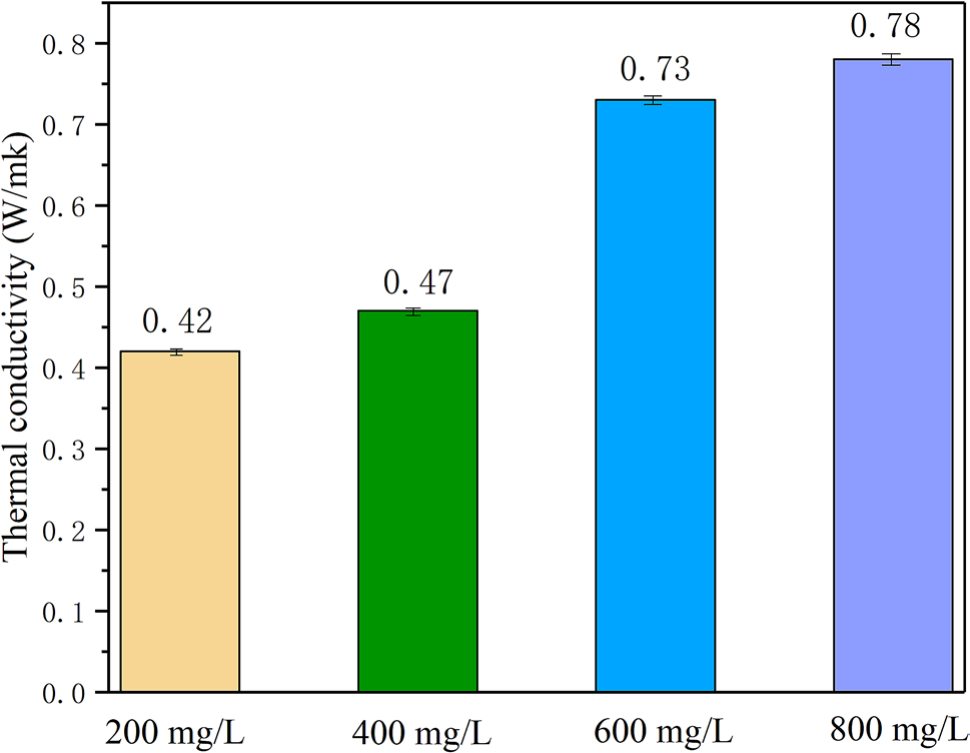
The thermal conductivity of 70% PEG/SMS-CSP at different AgNO_3_ concentration.

The concentration of AgNO_3_ in the preparation process of PEG/SMS-CSP possessed an obvious effect on the thermal conductivity of the ss-PCMs, and Fig. 15 showed the thermal conductivity of 70% PEG/SMS-CSP with different AgNO_3_ concentration. When the concentration of AgNO_3_ was 200 mg/L, it could be seen that the thermal conductivity of PEG/CSP was 0.42 W/(m K), and when the concentration of AgNO_3_ was 400 mg/L, the thermal conductivity of 70% PEG/SMS-CSP increased gently. When the AgNO_3_ concentration was 600 mg/L, the thermal conductivity of 70% PEG/SMS-CSP increased suddenly, which might be due to the fact that when the AgNO_3_ concentration was 400 mg/L, a small number of silver microspheres were obtained by in-situ reduction at 1300 ºC, and the silver microspheres could not contact with each other, resulting in the inhibition of electron transfer between silver microspheres particles, and leading to the decrease of the thermal conductivity of 70% PEG/SMS-CSP. When the AgNO_3_ concentration was 600 mg/L, more silver microspheres could be obtained, and the spherical structure of silver microspheres played a "bridging" role between PEG and porous-carbon, which provided a favorable heat flow channel and promoted the formation of thermal network and led to the rapid increase of thermal conductivity of the 70% PEG/SMS-CSP. When AgNO_3_ concentration was 800 mg/L, the thermal conductivity of 70% PEG/SMS-CSP could reach 0.78 W/(m K), which was increased by 300% compared to that of the pure PEG. It could be found that when the concentration of AgNO_3_ increased from 600 to 800 mg/L, the increment of the thermal conductivity of 70% PEG/SMS-CSP was slight. The reason was because when the concentration of AgNO_3_ was 600 mg/L, the adsorption amount of silver ions by cotton stalk porous-carbon was nearly saturated. Hence, when the concentration of AgNO_3_ increased to be 800 mg/L, the increment of the adsorption quantity of silver ions by CSP was small, and therefore, after in-situ reduction the amount of silver microspheres on the surface of CSP increased a little, resulting in a slight increase for the thermal conductivity of 70% PEG/SMS-CSP while the concentration of AgNO_3_ increased from 600 to 800 mg/L.

**Figure 16:**
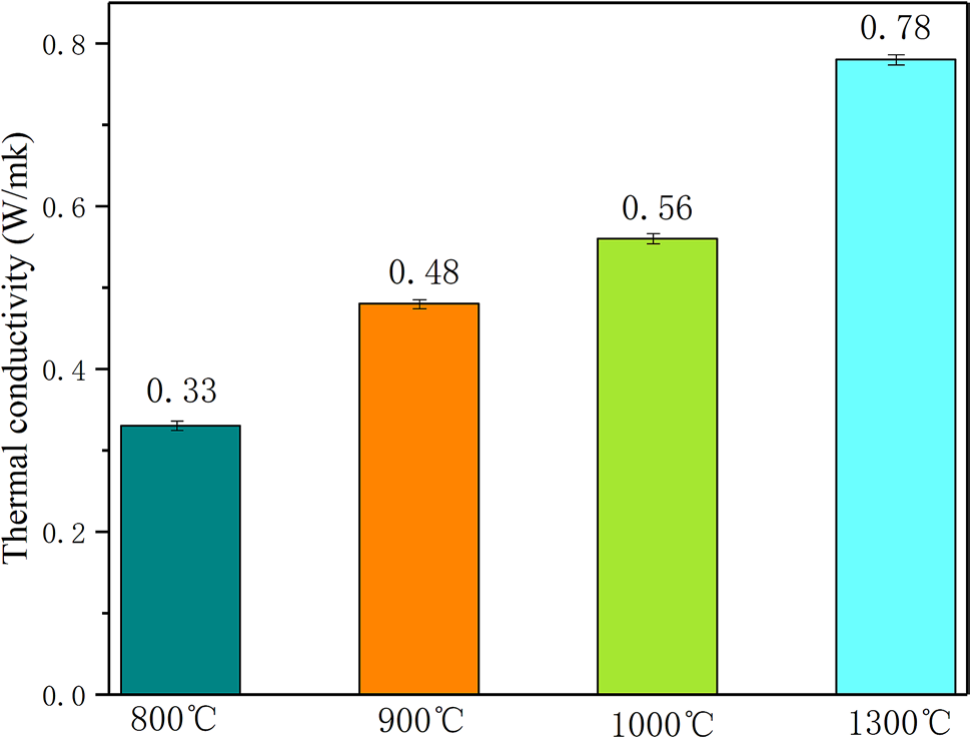
The thermal conductivity of 70% PEG/SMS-CSP at different pyrolysis temperatures.

Figure 16 shows the thermal conductivity of 70% PEG/SMS-CSP at different pyrolysis temperatures. It could be seen from Fig. 16 that the thermal conductivity of 70% PEG/SMS-CSP prepared at 800 °C was 0.31 W/(m K), and that of 70% PEG/SMS-CSP prepared at 1300 °C could reach 0.78 W/(m K), which might be due to the influence of pyrolysis temperature on the structure of porous-carbon. At high pyrolysis temperature, the original tube bundle structure of CSP was destroyed, and the wall of the hole collapsed, forming more channel structures. Therefore, the pore distribution of CSP pyrolyzed at 1300 °C was more uniform than that of CSP pyrolyzed at 800 °C, and the specific surface area of CSP pyrolyzed at 1300 °C could also be enlarged. The CSP with more uniform pore distribution could provide more heat transfer channels, and moreover, the graphitization degree of carbon was increased with the increase of pyrolysis temperature^32^. Therefore, the thermal conductivity of 70% PEG/SMS-CSP was enhanced with the increase of the pyrolysis temperature. Table 2 shows the comparison between the thermal properties of ss-PCMs prepared in the references and the 70% PEG/SMS-CSP developed in this study. It is clear that the thermal conductivity of 70% PEG/SMS-CSP was the highest. Additionally, the raw material of cotton stalk used to prepare CSP was agricultural residue, and the application of cotton stalk could make full use of agricultural wastes. Therefore, the 70% PEG/SMS-CSP prepared in this work possessed great application potentials.

**Table 2:** Comparison of the thermal properties of 70% PEG/SMS-CSP with other phase change materials prepared in previous studies.

Composite PCM	Melting process		Freezing process		Thermal conductivity (W/m K)	References
	△H_m_ (J/g)	T_m_ (°C )	△H_e_ (J/g)	T_e_ (°C)		
RHB/CO	70.08	22.85	65.10	33.03	0.20	^13^
LA/CC	199.60	37.50	148.30	35.10	0.44	^13^
LA/woods	177.90	–	178.20	–	0.27	^19^
PEG/WR	159.70	56.50	155.60	37.90	0.48	^33^
PEG/ASB	82.73	52.47	78.76	38.87	0.40	^17^
PEG/CMS-JPB	108.20	52.00	98.20	42.20	0.63	^20^
NPC/MA-SA	162.79	48.88	159.56	34.70	0.37	^34^
PAGFP	93.56	49.17	94.11	41.60	0.76	^35^
70% PEG/SMS-CSP	122.90	60.90	114.00	33.20	0.78	In this work

RHB/CO: rice husk/coconut oil. LA/CC: lauric acid/Carbonized corncob. PEG/WR: polyethylene glycol/white radish. PEG/ASB: polyethylene glycol/almond shell biochar. PEG/CMS-JPB: polyethylene glycol/copper microspheres and Jujube pit biochar. NPC/MA-SA: N-doped porous carbons Stearic acid and myristic acid. PAGFP: Porous Al_2_O_3_@graphite foams/paraffin.

According to the above study results, it was demonstrated that the non-uniformly dispersed spherical silver microspheres on the surface of CSP formed a thermal conductive path in the matrix, which improved the thermal conductivity of PEG/SMS-CSP dramatically. In addition, we found that the thermal conductivity of PEG/SMS-CSP could be improved by adjusting the structure of porous-carbon through changing the pyrolysis temperature, which provided more means for the thermal conductivity enhancement of ss-PCMs.

## Conclusion

In this study, a novel shape-stable phase change material of PEG/SMS-CSP was developed using silver microsphere doping porous-carbon as the matrix. The preparation conditions such as PEG content, pyrolysis temperature and AgNO_3_ concentration were optimized. The results showed that the maximum content of PEG in PEG/SMS-CSP was 70%, and with the increase of pyrolysis temperature and AgNO_3_ concentration, the thermal conductivity of PEG/SMS-CSP could be enhanced. The thermal conductivity of 70% PEG/SMS-CSP could reach 0.78 W/(m K), which was increased by 300% compared to the pure PEG, and the phase change enthalpy of 70% PEG/SMS-CSP was 122.9 J/g. Furthermore, the PEG/SMS-CSP possessed remarkable thermal stability and thermal reliability, and therefore, the PEG/SMS-CSP developed in this study could be applied as an ideal thermal energy storage material.
